# Volumetric CT Assessment of In Situ Induced Hepatic Lesions in a Transgenic Swine Model

**DOI:** 10.3390/life14111395

**Published:** 2024-10-30

**Authors:** Derek Smetanick, Danielle Stolley, David Fuentes, Natalie W. Fowlkes, Faith Shakoor, Maria Sophia Stenkamp, Samantha Hicks, Steve Parrish, Erik Cressman

**Affiliations:** 1 University of Arizona College of Medicine Tucson, Tucson, AZ 85724, USA; dereksmetanick@arizona.edu; 2 Department of Interventional Radiology, MD Anderson Cancer Center, The University of Texas, 1515 Holcombe Blvd., Houston, TX 77030, USA; dstolley@mdanderson.org (D.S.);; 3 Department of Imaging Physics, MD Anderson Cancer Center, The University of Texas, 1515 Holcombe Blvd., Houston, TX 77030, USA; 4 Department of Veterinary Medicine and Surgery, MD Anderson Cancer Center, The University of Texas, 1515 Holcombe Blvd., Houston, TX 77030, USAmsstenkamp@mdanderson.org (M.S.S.);

**Keywords:** hepatocellular carcinoma, large animal model, mathematical modeling, image segmentation

## Abstract

The growth rate of in situ-induced hepatic lesions in an Oncopig large animal model is quantitatively assessed. Oncopigs (n = 9) received baseline triple-phase CT scans prior to lesion induction. Lesions were subsequently induced by delivering the Ad-Cre vector to four locations in the liver. Triple-phase CT scans were obtained weekly to track the growth of the lesions. Animals were sacrificed at 14, 21, or 28 days (n = 3 in each group). The overall success rate of lesion generation was ~78%. Histopathology sections consistently revealed lesions that were highly inflammatory and consisted of a large leukocyte population without clear evidence of carcinomas. Lesions presented within imaging as hypovascular, low attenuating masses with slight contrast enhancement around the margins but little to no enhancement within the lesions themselves. The observed lesions were manually segmented on the venous phase image. Segmentation volumes were fitted to a logistic growth and decay model. Several lesions observed at earlier time points in the 28-day group had fully regressed by the time of the necropsy. The overall trend of rapid growth for the first 21 days, with spontaneous regression of the lesions being observed from day 21 to 28, suggests that the optimal window for experimental studies may be from days 14 to 21. The data and mathematical models generated from this study may be used for future computational models; however, the current model presented has moderate clinical relevance because many induced tumors resolved spontaneously within a few weeks. Awareness and careful consideration of the modest relevance and limitations of the model are advisable for each specific use case.

## 1. Introduction

Human hepatocellular carcinoma (HCC) is the most common primary liver cancer [1], the sixth most common cancer overall, and the third highest cause of cancer-related death [2,3]. Chronic liver inflammation can progress to fibrosis or cirrhosis and subsequently elevate the risk of developing hepatocellular carcinoma (HCC). Such inflammation may be due to factors such as chronic viral infections (hepatitis B or C), persistent alcohol consumption, and metabolic disorders [4,5]. With 90% of HCC cases developing in conditions of chronic inflammation, fibrosis, or cirrhosis, the immune system plays a major role in the tumor microenvironment [6].

Multidisciplinary management with guidance provided by treatment algorithms provides the foundation for the current standard of care for HCC. Transarterial Chemoembolization (TACE) is a procedure performed by interventional radiologists that is a first-line therapy for Barcelona Clinic Liver Cancer B/intermediate HCC in patients without vascular involvement [2,7,8]. Research into methods used for locoregional therapy in appropriate patients relies extensively on animal models. Rodent models have been used to study TACE [9,10], but the small size of their vessels hinders or prevents the use of catheter-based treatments in many cases [11]. In a lobar embolization study of rats, there was a high periprocedural mortality rate of 56%, with extensive tissue necrosis of the liver and lungs likely due to nontarget embolization [10]. Additionally, the genetic variations seen in human HCC are diverse, making it challenging for HCC models using inbred rodent strains to accurately mimic the molecular aspects of human HCC disease [12]. The rabbit VX2 model has been used for decades to test intraarterial therapies such as TACE, thermoablative therapies, and radioembolization [13]. However, there are substantial limitations of this model and its relevance, which can affect translatability to the clinic as well as drug dosing [14].

Swine models offer significant advantages in scale and relevance to humans but with trade-offs in cost and availability. Swine have been used for testing image-guided therapies given their similar size, anatomy, and physiology to humans [11]. Inducing carcinomas presents a challenge, however. Use of diethylnitrosamine (DEN), as often used in rodents, involves administering intraperitoneal injections of DEN over the course of 3 months and waiting up to 2 years for HCC to develop in a cirrhotic environment [15,16]. The drawbacks of this model are the length of time required (which adds considerably to the cost) and genetic variability caused by individual pigs’ reactions to DEN [11]. The recently developed Oncopig model has KRAS and TP53 mutations (oncogenic KRASG12D and dominant-negative TP53R167H), which are commonly found, particularly in cancers associated with the gastrointestinal tract. Genetic relevance for the Oncopig in HCC research is modest, however, as KRAS is not commonly mutated in HCC. Nevertheless, the pressing need for new therapies for HCC and the limitations of existing large animal models have led to the application of this model in translational research. Two equivalent approaches with the Oncopig model have been reported: the cell line approach and the in situ approach [17]. The cell line approach involves partial hepatectomy and cell isolation [18,19]. From this tissue sample, isolated hepatocytes are then transformed with Ad5CMVCre-eGFP and passaged in cell culture to then be percutaneously injected into the pig’s liver with a gelatin sponge [17].

The second method, known as the in situ approach, involves incubating a biopsy core sample of tissue with a solution of the adenoviral vector carrying the Cre recombinase gene [17]. The virus, gelatin sponge, and biopsy tissue are injected percutaneously back into the pig through the same guide cannula used for the biopsy. Quantitative data on growth curves using this model have not been previously reported. We address this gap, reporting the volumetric growth rate of hepatic lesions and histologic findings and developing a mathematical model describing the growth curves.

## 2. Results

### 2.1. Computational Modeling

**Figure 1:**
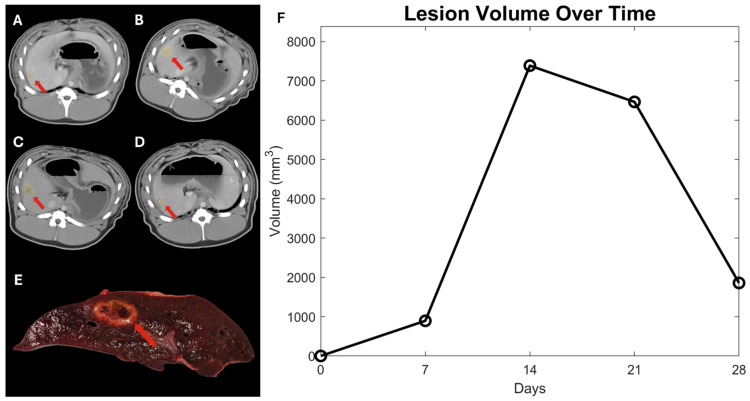
CT imaging from the longitudinal study is shown for a representative 28-day Oncopig with (**A**) imaging from 7 days, (**B**) 14 days, (**C**) 21 days, and (**D**) 28 days. The red arrows point to a hepatic nodule in the area of Ad-Cre injection. The tissue slice shown in (**E**) corresponds to the right lateral lesion visible in the CT imaging shown in d. The overall trend for this lesion was increasing volume until day 14, followed by regression from day 14 to day 28. (**F**) The time volume history is shown in time (days) and volume (mm^3^).

The in situ-induced liver tumors appeared as hypovascular, low attenuating masses with slight contrast enhancement around the margins but little to no enhancement within the tumors themselves on CT. CT demonstrated an in situ inoculation success rate of ~78%. However, several lesions present on CT imaging at earlier time points had fully regressed on imaging and necropsy at the 28-day time point. Figure 1 demonstrates a 28-day pig showing the imaging at 7, 14, 21, and 28 days for a lesion in the right lateral lobe. The lesion volume is plotted over time to show the growth trend of that specific lesion.

The rate of lesion growth varied widely between pigs, but the general trends remained relatively consistent within each individual pig. The volumetric data were fit to Equation (1), shown below, which was derived from Appendix A. MATLAB (Mathworks, Natick, MA, USA) lsqnonlin optimization function version 2022 [20] was used to optimize the values for C, κ, and θ.

(1) $$V=\frac{\theta }{146,997\cdot \left(1-C\cdot e^{-k\cdot t}\right)}$$

The values for C, κ, and θ were initialized to −10, 0.1 $\frac{1}{day}$, and 146,997 $\frac{cells}{\mu L}\cdot 10^{4}\;\mu L$, respectively, for the lesion growth. These values were chosen based on the values found in the prior literature [21,22]. Given that the data showed a lesion decay after 21 days, the κ value needed to be negative for the decay after three weeks. The values for C, κ, and θ were initialized to −10, −0.1 $\frac{1}{day}$, and 146,997 $\frac{cells}{\mu L}\cdot 10^{4}\;\mu L$, for this final week. After optimizing with max iterations set to 10,000, the optimized values for C, κ, and θ are tabulated in Table 1 below for both the period of lesion growth and the period of lesion regression.

**Figure 2:**
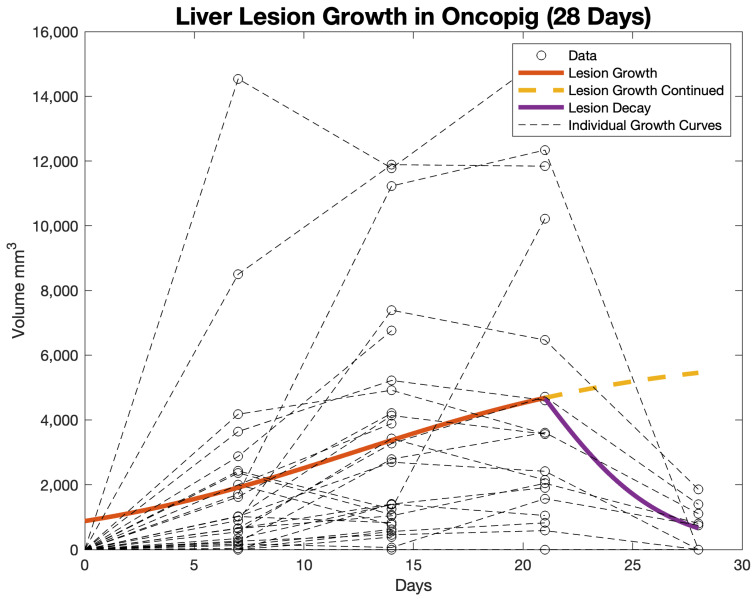
Computational model of the lesion growth and lesion decay using logistic growth and decay mathematical models.

The values for C, κ, and θ that appear in Table 1 were used to derive the lesion growth and lesion regression equations. These equations are graphed in Figure 2. The overall pattern for lesion growth showed an increase in volume up to day 21 at a linear rate of ~223.76 mm^3^/day, followed by regression between days 21 and 28 at a linear rate of ~573.1 mm^3^/day. However, given the logarithmic growth of tumors, these rates are not linear, and the mathematical modeling shows a more realistic growth and regression trend. The lesion growth was extrapolated for 7 days after the 21 days to show the disparity between what would be expected with predicted growth versus our hepatic lesion model, which had lesion regression. Individual tumor growth curves are plotted for comparison to the overall growth curve.

### 2.2. Pathology

**Figure 3:**
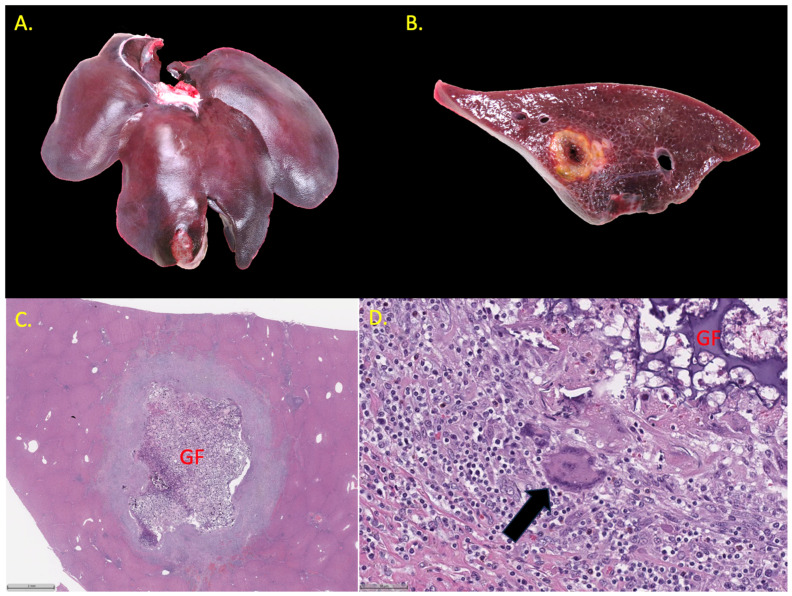
Ad-Cre-associated pathology in transgenic Oncopigs. (**A**) Image showing whole liver 21 days after Ad-Cre injection (Pig #21). (**B**) An intrahepatic, well-demarcated, pale tan nodule is present on the cut surface. (**C**) Photomicrograph showing well-demarcated hepatic nodule 21 days after Ad-Cre injection with gel foam (GF) (H&E, 1×). (**D**) Photomicrograph showing mixed inflammation, multinucleated giant cells (arrow), adjacent to gel foam (H&E, 20×).

**Figure 4:**
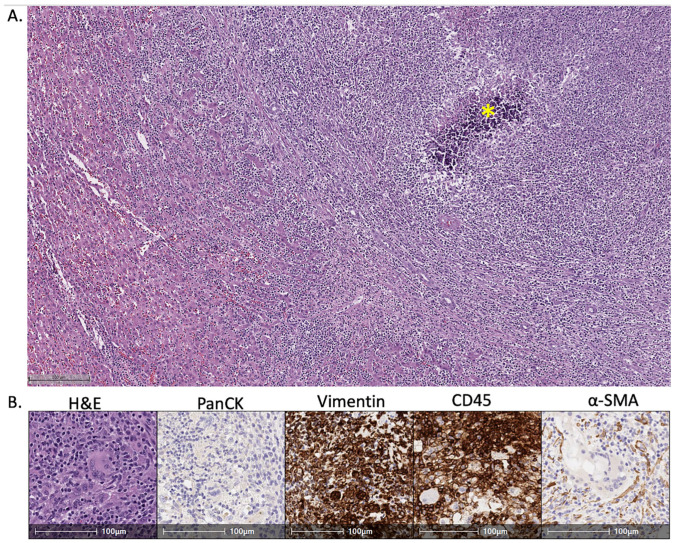
Immunohistochemical characterization of Ad-Cre-induced hepatic nodules in transgenic Oncopigs. (**A**) Photomicrograph showing dense cellular infiltration and dystrophic mineralization (*) in area of Ad-Cre injection (H&E, 5×). (**B**) Photomicrographs showing infiltration by immune cells (H&E, 20×), which are negative for pancytokeratin (no epithelial differentiation) and positive for vimentin and CD45 (mesenchymal differentiation and leukocyte markers). Scattered alpha smooth muscle actin-positive fibroblasts (myofibroblasts) are also present, supporting some element of a healing response.

At necropsy, areas injected with Ad-Cre varied in appearance at 14 days from superficial, tan, irregular, variably well-demarcated, roughened, 0.5–1.0 cm thick plaques on the peritoneal surface (peritonitis) to soft, variably well-demarcated nodules in the hepatic parenchyma that were typically characterized by a necrotic center around the gelfoam with a firm, tan rim of inflammation and/or fibroplasia at the periphery, as seen in Figure 3. At 21 days, lesions were similar except more discrete, firm, and well-demarcated. At 28 days, areas of hepatitis had increased fibrosis, and in some areas of Ad-Cre injection, all that remained was predominantly scar. Peritonitis was generally present to varying degrees at all time points and sometimes resulted in adhesions between the liver and the diaphragm. All lesions identified at necropsy consisted of mixed inflammation characterized by variable numbers of macrophages, lymphocytes, plasma cells, and neutrophils on histopathology, as shown in Figure 4; multinucleated giant cells were present in some areas especially associated with gelfoam. Mild biliary hyperplasia was present adjacent to some areas of intense inflammation, but carcinoma was not identified in any of the examined specimens on histopathology. This stands in contrast to Nurili et al. [17], who found poorly differentiated to undifferentiated carcinomas in addition to a major inflammatory component.

## 3. Discussion

Mathematical characterization of the growth rate of cancer is important for understanding tumor dynamics [23]. Logistic growth has been shown in the literature to be a good mathematical model for tumor growth [21]. Our results agree with the literature and demonstrate that the logistic model is a reasonable approximation to the population average tumor growth dynamics. Here, we focus on the average of the animal cohort in our study. Our curve fit provides a quantitative estimate of the lesion growth and regression that was observed experimentally and provides a reference for characterizing the growth dynamics of the Oncopig model of HCC. Further work may extend mathematical modeling to consider the animal-specific variations to characterize the tumor growth dynamics, cross-validate animal-specific parameters, and ultimately predict response to therapy [24,25,26].

Our mathematical results quantify a negative growth rate of −0.36 $\frac{1}{day}$ after week three. The negative growth rate has not been previously reported and is in contrast with prior published work. This highlights that several factors must be borne in mind for the successful application of the model. These include the need for a carcinoma per se, the relative importance of the immune response, the optimal window for performing an experiment, the duration of an experiment after the intervention, and whether or not the type of cell death is part of an investigation.

Based on our experience, the first question is whether the goals of a given project depend critically on the development of a carcinoma. This would apply in the case of a new chemotherapeutic agent, for example. If this is the goal, the model in its current iteration may not be well suited. To successfully conduct research along these lines, it would be essential to refine the induction protocol to ensure that it reproducibly induces carcinomas rather than the inflammatory pseudotumors observed in the present study. Longitudinal studies in large animal models for overall survival, as is common in mouse models, however, are associated with extremely high costs. As a result, such studies are rarely performed, and this use case may ultimately not prove to be a major issue.

The profound inflammatory response has been noted in previous work. It appears to be independent of the organ of interest, as it has been reported in the liver [17,27] and pancreas [28], even going so far as to cause fatal phlegmonous pancreatitis. The immune landscape of tumors describing the amount and activity of immune cells is informally referred to in the literature as a spectrum from cold to hot, with cold tumors having low infiltration and poorer prognosis than so-called hot tumors. A mass that consists exclusively or almost exclusively of immune cells would seem to be far beyond what would normally be considered hot. Thus, any research, particularly in immunotherapy with this model, would have to overcome a significant obstacle from the outset in terms of how to interpret data. Indeed, it becomes difficult to accurately assess whether the masses produced are an inherent response to the vector or if a carcinoma had been transiently induced, followed by an overwhelming immune response. As we did not identify carcinoma in any of the animals at any of the three time points (nine animals with four inoculations each), the latter seems less likely to be the case. While not a focus of the present report, the observed resolution over time could potentially be explained as the natural history of a resolving viral infection from a high-titer one-time dose of the vector.

If carcinomas are not essential to the research question but a discrete mass in the tissue is required, there are several areas that may benefit, and the model in its current form may aid in the clinical translation of technologies under investigation. One example is the development of robotics and in-training applications for surgery. In these areas, an actual tissue-based lesion brings an important element of fidelity to human surgical procedures that is absent in phantoms or artificial masses created in vivo [29,30]. Another application could be in the development of targeting software for ablation modalities such as histotripsy [31,32]. The model may also prove suitable for advancing new modalities such as augmented reality [33].

Our results indicate that careful timing of the experiments is critical to ensure a successful outcome for any experiments. For many of the scenarios discussed above, an identifiable lesion of suitable and consistent size is needed. In this respect, our findings are generally congruent with prior reports that adequate size is obtained within two weeks after inoculation. Coincident with timing is the question of duration. The data presented here indicate that the model would have the most value in an acute setting or very short-term subacute setting. This constraint is forced by the observed spontaneous regression that could confound the interpretation of results. Control groups such as the delivery of gelfoam alone and gelfoam with biopsy tissue but no vector would add strength to an ideal study design but be beyond the scope of the initial investigation. Inoculation in wild-type pigs has been previously reported and was not observed to provoke an inflammatory response [28].

In a similar vein, there is an interplay between the need for data concerning mechanisms for cell death and timing for endpoints in any proposed studies. Metrics for histology could be at risk of misinterpretation if such data are key to a particular project. Spontaneous central necrosis was observed in most tumors at necropsy. Thus, research in which necrosis was a dependent variable after intervention would, therefore, potentially be confounded by this result.

## 4. Materials and Methods

### 4.1. Animals

All in vivo work was performed under an IACUC-approved protocol. The technique was reported by Nurili et al. [17] and their work in describing the in situ method for tumor induction inside of the Oncopig. A brief description of the inoculation techniques is provided in Supplemental S1. For this study, 9 Oncopigs (transgenic pigs with Cre-inducible TP53R167H and KRASG12D mutations) were obtained (University of Illinois Urbana-Champaign). All procedures and imaging were performed under general anesthesia with peri-operative analgesia. Anesthesia induction was performed using tiletamine hydrochloride and zolazepam intramuscular injection (Zoetis US, Parsippany, NJ, USA). After intubation, anesthesia was maintained with 4% isoflurane to effect. Post-operative analgesia was accomplished with buprenorphine or meloxicam at the discretion of the veterinary staff. The ARRIVE guidelines were followed as shown in Supplemental S2.

### 4.2. In Situ Tumor Induction

The in situ tumor induction method in Oncopigs, as previously described in the literature [17], was utilized. Through a 17G guide cannula, an 18G liver biopsy core sample was obtained from each site in the Oncopig using the standard co-axial technique. The tissue was subsequently incubated with an adenoviral vector carrying the Cre recombinase gene (Gene Transfer Vector Core, University of Iowa, Iowa City, IA, USA). Gelatin sponge was incorporated into the mixture, which was then delivered back into the liver via the biopsy needle. Each Oncopig received a total of four liver inoculations distributed across the right and left lateral and medial lobes.

### 4.3. Imaging

Prior to inoculation, each animal underwent baseline triple-phase computed tomography (Siemens Healthineers, Forchheim, Germany). Following tumor induction, weekly triple-phase CT scans were acquired to monitor tumor growth. High-resolution images were obtained for a comprehensive assessment of induced tumors and surrounding liver tissue.

### 4.4. Image Segmentation

Due to the hypovascular nature of the lesions, the portal venous phase images were selected for analysis, as tumors were most conspicuous in this phase of imaging. The 14-day Oncopig cohort thus underwent imaging at three time points: baseline CT imaging prior to in situ tumor induction and subsequent scans at 7 and 14 days post-inoculation. The 21-day cohort received imaging at four intervals: baseline, 7, 14, and 21 days post-inoculation. Similarly, the 28-day cohort underwent five imaging sessions: baseline, 7, 14, 21, and 28 days post-inoculation. The final imaging for each pig occurred prior to euthanasia and necropsy.

Image segmentation was performed using 3D Slicer software version 5.2 (https://www.slicer.org/, accessed on 1 June 2023) [34]. Initially, the portal venous phase CT scan DICOM files were imported into 3D Slicer with the preset abdominal filter window used. Tumors were identified in the axial view of the CT images. The tumor was manually segmented in the segment editor tool. The island tool was used to eliminate extraneous structures, ensuring a continuous tumor mass. Segmentation accuracy was reviewed in each axial, coronal, and sagittal view under the supervision of an interventional radiologist (EC) with 20 years of experience. Segmentation statistics were calculated internally from 3D Slicer, which included voxel count, volume (mm^3^), and surface area (mm^2^). The volume data were used to determine hepatic lesion growth characteristics.

### 4.5. Mathematical Modeling

Using the volumetric data from CT, we can model the growth and regression of the tumors through Equation (2), adapted from Weiss et al. [21]

(2) $$\frac{\partial N}{\partial t}=k\cdot N\cdot \left(1-\frac{N}{\theta }\right)$$

where t is time in days, N is the number of cells in the lesion, κ is the spatially dependent growth rate ($\frac{1}{day}$), and θ is the tumor cell carrying capacity (number of tumor cells). Given our volumetric CT data, we can approximate the number of cells in a lesion using Equation (3) below.

**Figure 5:**
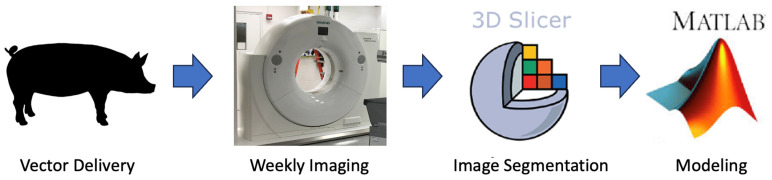
Study workflow demonstrating the progression from in situ injection to the development of mathematical models.

N = αV(3)

where V is volume in mm^3^, and α is 146,997 ± 15,738 $\frac{cells}{mm^{3}}$ in a normal swine liver parenchyma [22]. The volumetric data were fit to the differential equation and optimized using MATLAB’s nonlinear least squares solver [20]. Growth and regression were fit independently. A negative growth rate corresponds to regression. Growth was assumed between injection and 3 weeks. Regression was assumed in week 3 and beyond. The steps are further summarized in Figure 5.

### 4.6. Pathology

Pigs were euthanized at 14 (n = 3), 21 (n = 3), and 28 days (n = 3), and necropsy was performed. Representative sections of liver were collected and fixed in 10% neutral buffered formalin for 48–36 h. Tissues were processed routinely and paraffin embedded. Sections were cut at 4 µm using a microtome, placed on positively charged glass slides, and stained with hematoxylin and eosin (H&E). Immunohistochemical staining for pancytokeratin (Bioss, Woburn, MA, USA, cat. no. 1712R), vimentin (Cell Signaling Technologies, Danvers, MA, USA, cat. no. 5741), alpha smooth muscle actin (Abcam, Cambridge, UK, cat. no. 5694), and CD45 (Abcam, cat. no. 10558) were performed using a Leica Bond RXm autostainer (Deer Park, IL, USA). Slides were reviewed by an ACVP board-certified veterinary pathologist (NWF)

## 5. Conclusions

The induced liver tumors showed a general pattern of rapid growth during the first 21 days, followed by spontaneous regression between days 21 and 28, with some tumors completely regressing. Pathology indicated that these tumors are highly inflammatory. The CT volumetric data generated from this study suggest the optimal window spans 2–3 weeks after inoculation for treatments with immediate evaluation of endpoints before spontaneous regression confounds data interpretation. Given these findings, caution is warranted when assessing a specific use case for this transgenic model in its current iteration and further refinements of this model if using a percutaneous delivery technique may be needed. The mathematical model developed can be applied in future computational studies to test new treatment methods or optimize existing therapies.

## Figures and Tables

**Table 1:** MATLAB optimized variables using lsqnonlin(). Growth was optimized using CT volumetric data from 7, 14, and 21 days, and regression was optimized using data from days 21 and 28.

	Variable	Optimized Value
Lesion Growth	c	5.823
	κ	0.146
	θ	8.740 × 10^8^
Lesion Regression	c	5.909 × 10^−4^
	κ	−0.360
	θ	1.470 × 10^9^

## Data Availability

The raw data supporting the conclusions of this article will be made available by the authors upon request.

## Supplementary Materials

The following supporting information can be downloaded at: https://www.mdpi.com/article/10.3390/life14111395/s1.

## Appendix A

We provide the derivation of the analytical solution for reference. The partial differential equation below demonstrates logistic growth, where N is the number of hepatocytes in the lesion, k is the spatially dependent growth rate in $\frac{1}{day}$, and θ is the carrying capacity of the lesion in mm^3^.

(A1) $$\frac{\partial N}{\partial t}=k\cdot N\cdot \left(1-\frac{N}{\theta }\right)$$

Solving the differential equation below

(A2) $$\int \frac{\partial N}{N\cdot \left(1-\frac{N}{\theta }\right)}=\int k\cdot \partial t$$

Rewrite in the following form

(A3) $$\int \frac{\theta \cdot \partial N}{\left(\theta -N\right)\cdot N}=\int k\cdot \partial t$$

Integrate the left side of the equation

(A4) $$\int \frac{\theta \cdot \partial N}{\left(\theta -N\right)\cdot N}$$

Apply linearity

(A5) $$-\theta \cdot \int \frac{\partial N}{N\cdot \left(N-\theta \right)}$$

Now solving for

(A6) $$\int \frac{\partial N}{N\cdot \left(N-\theta \right)}$$

Partial fraction decomposition breaks down the expression

(A7) $$\int \left(\frac{1}{\theta \cdot \left(N-\theta \right)}-\frac{1}{N\cdot \theta }\right)\cdot \partial N$$

Apply linearity

(A8) $$\frac{1}{\theta }\cdot \int \frac{\partial N}{N-\theta }-\frac{1}{\theta }\cdot \int \frac{\partial N}{N}$$

Solving the expression

(A9) $$\frac{\ln\left(N-\theta \right)}{\theta }-\frac{\ln\left(N\right)}{\theta }+C$$

Factoring out $\frac{-1}{\theta }$

(A10) $$\frac{-1}{\theta }\cdot \left(\ln\left(N\right)-\ln\left(N-\theta \right)\right)+C$$

Plug in Equation (A10) back into Equation (A5)

(A11) $$-\theta \cdot \frac{-1}{\theta }\cdot \left(\ln\left(N\right)-\ln\left(N-\theta \right)\right)+C$$

Simplification

(A12) $$\left(\ln\left(\left|N\right|\right)-\ln\left(\left|N-\theta \right|\right)\right)+C$$

Now integrate the right-hand side of Equation (A3)

(A13) k⋅t+C

Plug Equations (A12) and (A13) into Equation (A3)

(A14) $$\ln\left(\left|N\right|\right)-\ln\left(\left|N-\theta \right|\right)=k\cdot t+C$$

Take the exponential of both sides

(A15) $$\frac{N}{N-\theta }=C\cdot e^{k\cdot t}$$

Inverse both sides

(A16) $$\frac{N-\theta }{N}=C\cdot e^{-k\cdot t}$$

The relationship of the number of cells to volume is shown below, where V is the volume of the lesion in mm^3^.

(A17) N=a⋅V

From Junatas et al. 2017 [22], the numerical density of all hepatocytes was 146,997 ± 15,738 $\frac{cells}{mm^{3}}$ of liver parenchyma.

(A18) $$N=146,997\frac{cells}{mm^{3}}\cdot V$$

Equation (A18) can be plugged into Equation (A16)

(A19) $$\frac{146,997\cdot V-\theta }{146,997\times V}=C\cdot e^{-k\cdot t}$$

Simplification

(A20) $$1-\frac{\theta }{146,997\times V}=C\cdot e^{-k\cdot t}$$

Simplification

(A21) $$\frac{\theta }{146,997\times V}=1-C\cdot e^{-k\cdot t}$$

Solving for V

(A22) $$V=\frac{\theta }{146,997\cdot \left(1-C\cdot e^{-k\cdot t}\right)}$$
