# Supplementary material for: Volumetric CT Assessment of In Situ Induced Hepatic Lesions in a Transgenic Swine Model
Source: Life (Basel). 2024 Oct 30;14(11):1395. doi: 10.3390/life14111395 (PMC11595248; doi:10.3390/life14111395)
Supplement: Supplementary file 1 [file life-14-01395-s001.zip › Supplemental S1.pdf]

Brief description of the inoculation technique, closely adapted from Nurili et al J Vasc Interv Radiol 2021 Vol. 32 Issue 4 Pages 510-517 e3
Accession Number: 33500185 PMCID: PMC8451249 DOI: 10.1016/j.jvir.2020.09.011.
Differences from the reported method were

1. replacing the biopsy gun with an 18G Bard Mission core gun (BD, Franklin Lakes, NJ) instead of an 18G Temno needle,
2. inoculating 4 sites instead of 2,
3. performance of all biopsy/inoculation procedures under CT guidance only (no ultrasound was used), and
4. the needle was removed after approximately 1 minute.

An 18-gauge core biopsy specimen of the liver was obtained under computed tomography (CT) or ultrasound guidance (1- or 2-cm core length; Temno Evolution; Merit Medical Systems, South Jordan, Utah) using coaxial technique (18 pigs). $\textit{TP53}^{R167H}$ and $\textit{KRAS}^{G12D}$ expression was induced by incubating the core biopsy specimen with an adenoviral vector carrying the Cre recombinase gene (10^9 pfu Ad5CMVCre-eGFP; University of Iowa Viral Vector Core, Iowa City, Iowa) for 20 minutes at room temperature in phosphate-buffered saline containing 15- mM calcium chloride (total fluid volume of 1 mL). Gelatin sponge (Surgifoam; Ethicon, Somerville, New Jersey) was then added using a 3-way stopcock, and the mixture (virus, core biopsy specimen, gelatin) was injected percutaneously back into the pig's liver through the biopsy needle, which was kept in place.
